# Comparison of Five Different Criteria for Diagnosis of Subclinical Hypothyroidism in a Large-Scale Chinese Population

**DOI:** 10.3389/fendo.2022.820414

**Published:** 2022-02-15

**Authors:** Yan-song Zheng, Sheng-yong Dong, Yan Gong, Jia-hong Wang, Fei Wang, Qiang Zeng

**Affiliations:** The Department of Health Medicine, Second Medical Center and National Clinical Research Center for Geriatric Diseases, Chinese People’s Liberation Army General Hospital, Beijing, China

**Keywords:** thyroid-associated hormones, hypothyroidism, subclinical hypothyroidism, diagnosis, criteria

## Abstract

**Background:**

Several different criteria for subclinical hypothyroidism (SCH) have been used in the literature, but the performance of these criteria was unknown.

**Objective:**

This retrospective study was to evaluate the diagnostic criteria for SCH.

**Methods:**

Eligible participants were based on centration of thyroglobulin antibodies (TG-Ab), thyroid peroxidase antibodies (TPO-Ab), and five thyroid-related hormones including total thyroxine (TT4), total triiodothyronine (TT3), free thyroxine (FT4), free triiodothyronine (FT3), and thyroid-stimulating hormone (TSH). Euthyroid individuals were identified *via* specific criteria. Five different SCH diagnostic criteria were compared based on the distributions of those indicators. An appropriate TSH cut-off value was reconsidered.

**Results:**

The study included 145,015 participants. The number of SCH cases diagnosed using criterion 5 was significantly different compared to the cases diagnosed using criteria 1-4 (*P*<0.05) and had the highest positive proportions of TG-Ab and TPO-Ab. Analysis of 60,515 subjects with normal other thyroid hormones revealed a median TSH concentration of 2.04 mIU/L, and the *P*_2.5_–*P*_97.5_ CI was 0.48-7.03 mIU/L. When the threshold for TSH elevation was elevated from ≥4.5 mIU/L to ≥6.50 mIU/L, the number of diagnosed SCH cases decreased from 7.30% to 2.09% and the proportions of positive TG-Ab and TPO-Ab increased from 23.69% and 24.07% to 33.75% and 35.06%, respectively (*P*<0.01).

**Conclusions:**

Combination of an elevated TSH and normal TT3, TT4, FT3, and FT4 concentrations is a must for the diagnosis of SCH. A new TSH threshold should be identified for better patient monitoring and management, according to the real-world characteristics of TSH distribution in Chinese population.

## Introduction

Subclinical hypothyroidism (SCH) is highly prevalent worldwide but remains challenging to diagnose. Individuals with SCH often do not have clinical symptoms and rarely seek medical care. However, several recent studies have shown that SCH is associated with coronary heart disease, hypertension, ischemic cerebrovascular disease, metabolic syndrome, osteoporosis, obstetric complications, and other diseases (1–3). Thus, attention has been drawn to identifying SCH, especially during physical examinations. It is generally accepted that SCH is characterized by elevated serum concentrations of thyroid-stimulating hormone (TSH) in the absence of clinical symptoms and thyroid hormone changes. However, due to technological limitations and the cost of screening, no consensus has been reached on how to diagnose SCH. For example, China’s Guidelines for the Diagnosis and Treatment of Adult Hypothyroidism define SCH as an endocrine syndrome associated with elevated concentrations of TSH but normal concentrations of serum total thyroxine (TT4) and serum free thyroxine (FT4) in the absence of obvious signs and symptoms (4). Other studies have defined SCH as a condition with elevated serum concentrations of TSH but normal concentrations of FT4 and free triiodothyronine (FT3) (5, 6). Another study has suggested diagnosing SCH based on elevated TSH concentrations and normal FT4 concentrations (7), while still other studies have recommended diagnosing SCH based on elevated TSH concentrations and normal serum concentrations of TT4 and serum total triiodothyronine (TT3) (8–11). Moreover, there are substantial differences in the reported cut-off values for identifying elevated TSH concentrations to diagnose SCH (12). These discrepancies have generated controversies regarding the diagnosis and clinical significance of SCH, and it is important to determine how to best diagnose SCH based on five to seven thyroid function indicators. This study aimed to compare different criteria for diagnosing SCH based on thyroid function indicators.

## Materials and Methods

We evaluated individuals who underwent physical examinations in the Chinese People’s Liberation Army General Hospital between March 2014 and November 2019. Subjects were considered eligible if they were ≥18yrs old and had undergone testing to evaluate thyroid function indicators. However, subjects were excluded based on missing data regarding sex, age, and laboratory findings for at least one of the five thyroid function indicators. In order to find out the real-world distribution characteristics of TSH in population with normal TT3, TT4, FT3, FT4, TSH, TG-Ab, and TPO-Ab, a specific group was identified with strict criteria, which was selected by excluding pregnant women; individuals with definite thyroid-related diseases or a history of thyroid-related surgeries; individuals with a thyroid nodule diameter of ≥1.0 cm; and individuals with abnormal results of thyroid-associated hormones except TSH, and with abnormal thyroglobulin antibodies (TG-Ab) and thyroid peroxidase antibodies (TPO-Ab), or thyroid ultrasonography.

The retrospective study protocol was approved (S2017-003-02) by the Chinese People’s Liberation Army General Hospital ethics committee and complied with the principles of the Declaration of Helsinki and its contemporary amendments.

### Data Collection

Data regarding age and sex were collected from the subjects’ medical records. Medical histories were collected *via* face-to-face interviews. The height, weight, and blood pressure were measured by well-trained nurses and doctors. The body mass index (BMI) was calculated as the weight divided by the height squared. All blood samples were collected after the subjects fasted for 8–12 hr. According to the quality control and testing standards set by the Clinical Laboratory Department of the Clinical Laboratory Department of the Chinese People’s Liberation Army General Hospital (13, 14), Fasting blood glucose, AST, triglyceride (TG), total cholesterol (TC), LDL-C and HDL-C levels were measured using a Roche C8000 automatic biochemical analyzer (Roche, Mannheim, Germany) with the corresponding reagents, calibrators, and quality control materials. The concentrations of TT4, TT3, FT4, FT3, TG-Ab, TPO-Ab, and TSH were determined using Roche Diagnostic reagents and the ACS:180 automatic chemiluminescence immunoassay system. The laboratory’s results had an intra-assay difference of <5% and an inter-assay difference of <10%. The normal reference ranges were 66.00–181.00 nmol/L for TT4, 1.30–3.10 nmol/L for TT3, 3.10–6.80 pmol/L for FT3, 12.00–22.00 pmol/L for FT4, <115.00 IU/mL for TG-Ab, <34.00 IU/mL for TPO-Ab, and 0.10–4.50 mIU/L for TSH.

Thyroid ultrasonography was performed by trained operators who were not aware of the laboratory test results. Thyroid size and morphology were evaluated using a high-resolution 7–13 MHz linear transducer, with the subject seated and their neck slightly extended.

### Age Grouping

We divided the subjects into three age-based groups: young subjects (<40yrs), middle-aged subjects (40–59yrs), and elderly subjects (≥60yrs). For some analyses, we created the following age stratification: <20yrs, 20–29yrs, 30–39yrs, 40–49yrs, 50–59yrs, 60–69yrs, 70–79yrs, and ≥80yrs.

### Statistical Analysis

Data were collected and analyzed using Stata software (version 11.0). Continuous data were reported as mean ± SD and categorical data were reported as number (%). The laboratory data for the thyroid-associated hormones were used to calculate the median value and confidence intervals (CIs) spanning the 5th to 95th percentiles (*P*_5.0_–*P*_95_ CIs) or the 2.5th to 97.5th percentiles (*P*_2.5_–*P*_97.5_ CIs). The variables were analyzed using the Kruskal-Wallis test, Wilcoxon rank-sum test, or chi-squared (χ^2^) test, as appropriate. Differences were considered statistically significant at *P*-values of <0.05.

## Results

### Demographic Characteristics

Between August 2013 and January 2018, a total of 150,035 subjects underwent physical examinations at the Chinese People’s Liberation Army General Hospital and were considered eligible. However, 5,020 subjects were excluded because they fulfilled the exclusion criteria. Therefore, the study ultimately evaluated 145,015 participants, including 90,011 male subjects (62.07%) and 55,004 female subjects (37.93%) with a mean age of 47.96 ± 9.72 yrs. The subjects were from 34 province-level administrative regions, which consisted of provinces, autonomous regions, directly controlled municipalities, and special administrative regions.

### Distributions of the Serological Thyroid Function Indicators

Normality tests indicated non-normal distributions for the concentrations of TT3, TT4, FT3, FT4, TSH, TG-Ab, and TPO-Ab. The median values and proportions of subjects with abnormal concentrations (relative to the reference ranges) were shown in **Table 1**. The highest abnormal rate was observed for TSH (10.21%), which was followed by TT3 (6.08%), FT4 (3.10%), TT4 (2.46%), and FT3 (1.01%). The median TSH concentration was 2.05 mIU/L, the *P_5.0_*–*P_95.0_* CI was 0.78–5.42 mIU/L, and the *P*_2.5_–*P*_97.5_ CI was 0.21–9.35 mIU/L. TSH results above the normal reference value (>4.5 mIU/L) were observed for 8.69% of the subjects. There was no significant difference in the proportions of subjects who were TG-Ab-positive and TPO-Ab positive (χ^2^ = 0.61, *P* = 0.43).

**Table 1:** Distributions of serological thyroid function indicators for the 145,015 subjects.

Variables*	Median value (P_2.5_-P_97.5_ Cis)	Below the normal range (%)	In the normal range (%)	Above the normal range (%)
**TT3 (nmol/L)**	1.71 (1.11–2.60)	8,408 (5.80)	136,201 (93.92)	406 (0.28)
**FT3 (pmol/L)**	4.90 (3.48–6.67)	327 (0.23)	143,553 (98.99)	1,135 (0.78)
**TT4 (nmol/L)**	97.63 (60.47–149.60)	3,276 (2.26)	141,454 (97.54)	285 (0.20)
**FT4 (pmol/L)**	16.21 (11.43–22.59)	2,533 (1.75)	140,526 (96.90)	1,956 (1.35)
**TSH (mIU/L)**	2.05 (0.21–9.35)	2,205 (1.52)	130,210 (89.79)	12,600 (8.69)
**TG-Ab (IU/mL)**	14.60 (10.00–789.10)		130,822 (90.21)	14,193 (9.79)
**TPO-A b (IU/mL)**	9.80 (5.00–543.00)		131,000 (90.34)	14,015 (9.66)

*All indices are presented as the median (interquartile range); Normal range: referred by the Roche diagnostic reagents instructions.

TT4, total thyroxine; TT3, total triiodothyronine; FT4, free thyroxine; FT3, free triiodothyronine; TSH, thyroid-stimulating hormone; TG-Ab, thyroglobulin antibodies; TPO-Ab, thyroid peroxidase antibodies.

### Diagnosing SCH According to the Different Diagnostic Criteria

When an elevated TSH concentration was defined as ≥4.50 mIU/L, we compared the diagnosis of SCH based on five different criteria: (1) an elevated TSH concentration with a normal FT4 concentration, (2) an elevated TSH concentration with normal concentrations of FT3 and FT4, (3) an elevated TSH concentration with normal concentrations of TT3 and TT4, (4) an elevated TSH concentration with normal concentrations of FT4 and TT4, and (5) an elevated TSH concentration with normal concentrations of TT3, TT4, FT3, and FT4. The results of the SCH diagnoses using these criteria are summarized in **Table 2**. There were no significant differences in the numbers of SCH cases diagnosed using criteria 1–4 (*P* > 0.05), although a significantly different number of SCH cases was diagnosed using criterion 5 (vs. criteria 1–4, *P* < 0.05).

**Table 2:** The different criteria for diagnosing SCH based on TSH concentrations of ≥4.5 mIU/L.

	Diagnosed SCH cases (%)	TG-Ab	TG-Ab	TPO-Ab	TPO-Ab
		Negative (%)	Positive (%)	Negative (%)	Positive (%)
**Criterion 1**	11,880 (7.92)	9,257 (77.92)	2,623 (22.08)*	9,214 (77.56)	2,666 (22.44)^†^
**Criterion 2**	11,814 (7.87)	9,204 (77.91)	2,610 (22.09)*	9,162 (77.55)	2,652 (22.45)^†^
**Criterion 3**	11,715 (7.81)	9,033 (77.11)	2,682 (22.89)*	9,068 (77.41)	2,647 (22.59)^†^
**Criterion 4**	11,622 (7.75)	9056 (77.92)	2,566 (22.08)*	9,021 (77.62)	2,601 (22.38)^†^
**Criterion 5**	10,948 (7.30)^‡^	8,354 (76.31)	2,594 (23.69)*^‡^	8,313 (75.93)	2,635 (24.07)^† ‡^

*Significantly higher proportion of TG-Ab positivity relative to the normal reference range criteria (9.79%), (P<0.01).

^†^Significantly higher proportion of TPO-Ab positivity relative to the normal reference range criteria (9.66%), (P<0.01).

^‡^Significantly different relative to the other diagnostic criteria (P<0.05).

SCH, subclinical hypothyroidism; TSH, thyroid-stimulating hormone; TG-Ab, thyroglobulin antibodies; TPO-Ab, thyroid peroxidase antibodies; Criteria 1, an elevated TSH concentration with a normal FT4 concentration; Criteria 2, an elevated TSH concentration with normal concentrations of FT3 and FT4; Criteria 3, an elevated TSH concentration with normal concentrations of TT3 and TT4; Criteria 4, an elevated TSH concentration with normal concentrations of FT4 and TT4; Criteria 5, an elevated TSH concentration with normal concentrations of TT3, TT4, FT3, and FT4.

Relative to criterion 5, criterion 1 identified an additional 932 SCH cases, which included 35 cases with decreased FT3 concentrations, 31 cases with increased FT3 concentrations, 256 cases with decreased TT4 concentrations, 2 cases with increased TT4 concentrations, 778 cases with decreased TT3 concentrations, and 5 cases with increased TT3 concentrations. Relative to criterion 5, criterion 2 identified an additional 866 SCH cases, which included 747 cases with decreased TT3 concentrations, 3 cases with increased TT3 concentrations, 253 cases with decreased TT4 concentrations, and 2 cases with increased TT4 concentrations. Relative to criterion 5, criterion 3 identified an additional 767 SCH cases, which included 7 cases with decreased FT3 concentrations, 32 cases with increased FT3 concentrations, 690 cases with decreased FT4 concentrations, and 44 cases with increased FT4 concentrations. Relative to criterion 5, criterion 4 identified an additional 674 SCH cases, which included 33 cases with decreased FT3 concentrations, 30 cases with increased FT3 concentrations, 638 cases with decreased TT3 concentrations, and 3 cases with increased TT3 concentrations. Thus, using criteria 1–4 resulted in varying numbers of misdiagnosed SCH cases, relative to criterion 5 (i.e., normal TT3, TT4, FT3, and FT4 concentrations with elevated TSH concentrations). Regardless of the diagnostic criterion that was used, significantly higher proportions of TG-Ab positive and TPO-Ab positive individuals were observed among subjects who were diagnosed with SCH with criterion 5 (*P* < 0.01). Moreover, both of the positive proportions of TG-Ab and TPO-Ab in criteria 5 were significantly higher than those in criteria 1–4 (*P* < 0.001).

### Identifying an Appropriate TSH Cut-Off Value

The strict criteria were fulfilled in 60,515 individuals, forming the specific group. Demographic characteristics of the individuals in this group were shown in **Table 3**. Those individuals showed normal concentrations of TT3, TT4, FT3, FT4, TG-Ab, TPO-Ab, and met other criteria, which included 43,357 males (71.65%) and 17,158 females (28.23%). The median TSH concentration was 2.04 mIU/L, the *P*_5.0_–*P*_95.0_ CI was 0.84–4.79 mIU/L, and the *P*_2.5_–*P*_97.5_ CI was 0.48-7.03 mIU/L in the special group. Thus, at least 5% of the 60,515 subjects had TSH concentrations that were ≥4.79 mIU/L. The Wilcoxon rank-sum test revealed a significant difference in TSH concentrations between male and female subjects (Z = 28.44, *P* < 0.001) (**Table 4**).

**Table 3:** Demographic characteristics of the individuals included in the specific group (n=60,515).

Variables	Total	Female (n=17,158)	Male (n=43,357)	Female vs Male
**Age (yrs)**	45.37 ± 9.23	44.15 ± 10.25	45.86 ± 8.75	z = 18.60, P = 0.009
**BMI (kg/m^2^)**	24.98 ± 3.54	22.83 ± 3.31	25.83 ± 3.25	z = 97.08, *P <*0.001
**SBP (mmHg)**	120.44 ± 16.59	112.81 ± 16.55	123.45 ± 15.61	z = 81.94, *P <*0.001
**DBP (mmHg)**	79.78 ± 11.69	75.44 ± 10.48	82.07 ± 11.43	z = 88.89, *P <*0.001
**Hb (g/L)**	149.22 ± 15.31	132.19 ± 11.77	155.47 ± 10.72	z = 232.75, *P <*0.001
**AST(U/L)**	21.04 ± 13.62	18.57 ± 8.76	21.79 ± 13.96	z = 73.70, *P <*0.001
**TC (mmol/L)**	4.76 ± 0.91	4.75 ± 0.91	4.76 ± 0.93	z = 4.42, *P <*0.001
**TG (mmol/L)**	1.82 ± 1.54	1.27 ± 0.89	2.04 ± 1.64	z = 113.03, *P <*0.001
**HDL-C(mmol/L)**	1.25 ± 0.34	1.46 ± 0.35	1.16 ± 0.28	z = 134.31, *P <*0.001
**LDL-C(mmol/L)**	3.03 ± 0.80	2.99 ± 0.81	3.07 ± 0.81	z = 17.82, *P <*0.001
**FBG (mmol/L)**	5.68 ± 1.35	5.39 ± 1.07	5.89 ± 1.51	z = 76.30, *P <*0.001
**Smoke**				χ2 = 12,000, *P <*0.001
** No**	34,248	15,851 (92.38%)	18,397 (42.43%)	
** yes**	26,267	1,307 (7.62%)	24,960 (57.57%)	
**Hypertension**				χ2 = 3,200, *P <*0.001
** No**	40,748	14,472 (84.35%)	26,276 (60.60%)	
** yes**	19,767	2686 (15.65%)	17,081 (39.40%)	

BMI, Body mass index; SBP, systolic blood pressure; DBP, Diastolic pressure; TC, Total cholesterol; TG, Triglyceride; HDL-C, HDL cholesterol; LDL-C, LDL cholesterol; FBG, Fasting blood glucose.

**Table 4:** Distributions of TSH concentrations in the different stratification in the specific group (n=60,515).

	N	Median TSH (mIU/L)	TSH (mIU/L) (P_5.0_-P_95.0_ CIs)	TSH (mIU/L) (P_2.5_-P_97.5_ CIs)	
**age stratification**					
** <20yrs**	115	2.17	0.98–5.37	0.65–6.01	χ2 = 62.42, *P* < 0.001
** 20–29yrs**	3,465	2.15	0.92–4.76	0.61–6.69	
** 30–39yrs**	10,554	1.98	0.87–4.49	0.55–6.34	
** 40–49yrs**	27,049	2.03	0.85–4.73	0.47–6.90	
** 50–59yrs**	15,862	2.05	0.81–4.96	0.41–7.39	
** 60–69yrs**	3,030	2.11	0.78–5.35	0.43–8.56	
** 70–79yrs**	421	2.11	0.76–6.29	0.44–9.23	
** ≥80yrs**	19	2.19	0.75–12.26	0.76–12.26	
**Age subgroups**					χ2 = 15.56, *P <*0.001
** Young subjects (<40yrs)**	14,134	2.04	0.88–4.55	0.57–6.49	
** Middle-aged subjects (40–59yrs)**	42,911	2.03*	0.83–4.82	0.45–7.09	
** Elderly subjects (≥60yrs)**	3,470	2.11*^†^	0.78–5.44	0.43–8.96	
**Sex stratification**					Z = 28.44, *P <*0.001
** males**	43,357	1.97	0.83-4.51	0.51-6.54	
** females**	17,158	2.26	0.88-5.44	0.37-8.09	
**Total**	60,515	2.04	0.84–4.79	0.48-7.03	

*Compared with young subjects; ^†^Compared with middle-aged subjects.

TSH, thyroid-stimulating hormone; Cis, confidence intervals.

The TSH concentration distributions for each age group were shown in **Table 4**. The Kruskal-Wallis test revealed significant differences in the TSH concentrations across the different age stratification (χ^2^ = 62.42, *P* < 0.001). Furthermore, there were significant differences when the subjects were grouped as young subjects, middle-aged subjects, and elderly subjects (χ^2 =^ 15.56, *P* < 0.001). The Wilcoxon rank-sum test also revealed significant differences between young and middle-aged subjects (Z=1.99, *P*=0.046), between young and elderly subjects (Z = 4.69, *P* < 0.001), and between middle-aged and elderly subjects (Z = 6.82, *P* < 0.001).

The *P*95th upper limits for TSH concentration were >4.50 mIU/L in all age groups, increased according to age, and reached 12.26 mIU/L for subjects who were ≥80yrs old. The *P*_2.5_–*P*_97.5_ CI values were 0.57–6.49 mIU/L for young subjects, 0.45–7.09 mIU/L for middle-aged subjects, and 0.43–8.96 mIU/L for elderly subjects. Thus, at least 2.5% of the 60,515 subjects with normal thyroid function and no thyroid-related diseases had TSH concentrations of ≥6.49 mIU/L.

There is general consensus that SCH patients with TSH concentrations of <10.00 mIU/L do not require clinical intervention (4, 15, 16), and that overdiagnosis could cause unnecessary psychological and economic burden. But the SCH patients with TSH concentrations of <10.00 mIU/L could develop clinical thyroid diseases every year, which means they could not be ignored and need monitoring. Therefore, although sex and age stratification were significant, we defined the TSH threshold for diagnosing SCH as 6.50 mIU/L as a clear, easily remembered, and practical cut-off value, according to the real TSH distribution in Chinese population, especially based on the Roche platform.

### Re-Diagnosis According to the New TSH Cut-Off Value

The new cut-off value for elevated TSH concentrations was applied to diagnostic criteria 1–5 in order to re-diagnose SCH and the results were summarized in **Table 5**. There were still no statistically significant differences in the number of SCH cases diagnosed using criteria 1–4 (*P* > 0.05), although a significantly different number of SCH cases was diagnosed using criterion 5 (vs. criteria 1–4, *P* < 0.05). Similarly, both of the positive proportions of TG-Ab and TPO-Ab in criteria 5 were significantly higher than those in criteria 1–4 (*P* < 0.01). Furthermore, the number of SCH based on criterion 5 and the new TSH threshold (≥6.50 mIU/L) was significantly less than that a diagnosis was based on a frequently-used TSH threshold of ≥4.50 mIU/L (2.09% vs. 7.30%; χ^2^ = 4,600, *P* < 0.001). Moreover, relative to a diagnosis using a TSH threshold of ≥ 4.50 mIU/L, SCH cases diagnosed using criteria 1–5 and the new threshold (≥ 6.50 mIU/L) had significantly higher proportions of TG-Ab-positive and TPO-Ab-positive cases (*P* < 0.01).

**Table 5:** Diagnosis of SCH based on the diagnostic criteria and TSH concentration threshold of ≥6.5 mIU/L.

	Diagnosed SCH cases (%)	TG-Ab	TG-Ab	TPO-Ab	TPO-Ab
		Negative (%)	Positive (%)	Negative (%)	Positive (%)
**Criterion 1**	3,466 (2.31)	2,421 (69.85)	1,045 (30.15)*	2,387 (68.87)	1,079 (31.13)*
**Criterion 2**	3,444 (2.30)	2,405 (69.83)	1,039 (30.17)*	2,371 (68.84)	1,073 (31.16)*
**Criterion 3**	3,594 (2.4)	2,380 (66.22)	1,214 (33.78)*	2,471 (68.75)	1,123 (31.25)*
**Criterion 4**	3,367 (2.24)	2,354 (69.91)	1,013 (30.09)*	2,321 (68.93)	1,046 (31.07)*
**Criterion 5**	3,132 (2.09)^†^	2,075 (66.25)	1057 (33.75)*^‡^	2,034 (64.94)	1098 (35.06)*^‡^

*Significantly different relative to a TSH concentration threshold of ≥4.5 mIU/L (P<0.01).

^†^Significantly different relative to the other diagnostic criteria (P<0.05).

^‡^Significantly different relative to the other diagnostic criteria (P<0.001).

SCH, subclinical hypothyroidism; TSH, thyroid-stimulating hormone; TG-Ab, thyroglobulin antibodies; TPO-Ab, thyroid peroxidase antibodies; Criteria 1, an elevated TSH concentration with a normal FT4 concentration; Criteria 2, an elevated TSH concentration with normal concentrations of FT3 and FT4; Criteria 3, an elevated TSH concentration with normal concentrations of TT3 and TT4; Criteria 4, an elevated TSH concentration with normal concentrations of FT4 and TT4; Criteria 5, an elevated TSH concentration with normal concentrations of TT3, TT4, FT3, and FT4.

## Discussion

There is significant controversy regarding the diagnosis and management of SCH (17, 18). Although an increasing number of studies have demonstrated that SCH has adverse effects on various physical functions, there are inconsistent diagnostic criteria for SCH. This has generated confusion in and unnecessary burden on the medical community (19). Our study compared five commonly used criteria for diagnosing SCH and revealed that criteria 1–4 were basically equivalent in terms of the number of diagnosed SCH cases, in which included some cases that obviously did not meet the definition of SCH. However, a significantly different number of cases was diagnosed using criterion 5 and the positive proportions of TG-Ab and TPO-Ab were significantly higher than those in criteria 1–4 (*P* < 0.001). Our results indicate that criterion 5 was stricter and avoided misdiagnosis of SCH. Therefore, we recommend using criterion 5 in the clinical setting based on the Roche platform, despite it being associated with an increased cost of SCH screening.

Clinical laboratories typically use the reference ranges that are proposed by test manufacturers. However, those ranges are generally based on data from non-Chinese populations and there has been no comprehensive assessment of the five thyroid-associated hormones and their relationships with thyroid disease in a large cohort of healthy Chinese individuals (11). Our study revealed that at least 5% of the Chinese population with completely normal TT3, TT4, FT3, FT4, TG-Ab, TPO-Ab and no thyroid-related diseases would be expected to have TSH concentrations of ≥4.50 mIU/L (the upper limit of the normal reference range). Moreover, both the upper 95.0^th^ and 97.5^th^ percentile values for TSH concentration increased with age and reached 12.26 mIU/L among individuals who were ≥80 yrs. As the purpose of a clinical diagnosis is to guide careful monitoring or intervention for the patient, a diagnosis that does not prompt additional steps has limited clinical significance and may increase the economic and psychological burden on the patient. Most recent studies indicate that drug intervention is not necessary for patients with SCH until they have TSH concentrations of ≥10.00 mIU/L. Therefore, we set the TSH threshold to ≥6.50 mIU/L. In Korea, also in Asia, park WR, et al. (20) found that when SCH was more predictive of overt hypothyroidism, the cut off value was TSH > 7.45 µIU/ml and higher prevalence positive anti-thyroid peroxidase (anti-TPO Ab) and anti-thyroglobulin antibody (anti-Tg Ab). But their study enrolled only 197 patients.

Interestingly, a diagnosis of SCH based on criterion 5 and the new TSH threshold (≥6.50 mIU/L) was made in a significantly smaller proportion of the study population, relative to when the diagnosis was based on a threshold of ≥4.50 mIU/L. However, the higher TSH threshold identified SCH in a smaller proportion of subjects, these cases had significantly higher proportions of TG-Ab and TPO-Ab positivity, relative to cases that were identified using the lower threshold. Previous studies have also shown that the incidences of clinical hypothyroidism and SCH are significantly higher when subjects have high titers of TPO-Ab (20) or test positive for both TG-Ab and TPO-Ab (21). Therefore, given that individuals who were diagnosed with SCH using the new TSH threshold (≥6.50 mIU/L) are more likely to clinically progress, they are more suitable for clinical monitoring or intervention. In Huber’s study (22), it was considered that TSH≥6 was more predictive in patients with SCH. In another neighboring Asian country, a cohort study in Japan found that TSH level >8 mIU/L was a predictive value for development of overt hypothyroidism (23). While a study in South Korea, which is also located in East Asia, found that the TSH threshold of 6.86 is more suitable for the diagnosis of SCH (24). TSH could vary in different countries or ethnic groups (25, 26). Therefore, our results at TSH were slightly different from those of other studies.

One limitation of our study is that it was a single-center study of Chinese adults. Thus, the results cannot be generalized to other racial and ethnic groups. In addition, TSH secretions are theoretically sensitive to very small changes in serum FT4, which means that TSH changes could occur during early-stage hypothyroidism even before FT4 abnormalities are detectable (27). However, in practice, TSH concentrations are influenced by numerous factors, including age, race, sex (28), acute illness, renal insufficiency, medications (29), pregnancy (30), depression (31), and anorexia nervosa (32). Meanwhile, between-assay differences and variations in reference ranges can directly impact the diagnosis and management of subclinical hypothyroidism (33). Therefore, changes in the TSH concentrations for our study may not have always been directly related to changes in the concentrations of TT3, TT4, FT3, or FT4, and may have even exhibited inverse relationships. However, our study also had several strengths. First, we used broad inclusion criteria, without excluding patients with unrelated diseases or medications, which presumably allowed us to capture both healthy people and people with subclinical thyroid disease. Therefore, our study sample is likely representative. Our study also used an ultra-sensitive TSH measurement technique to minimize the risk of indirect changes in TSH concentrations.

In conclusion, our study revealed that the diagnosis of SCH can be made based on elevated TSH concentrations in the presence of normal TT3, TT4, FT3, and FT4 concentrations (criterion 5). Other commonly used simplified criteria (criteria 1–4) were associated with an increased risk of misdiagnosis, based on the Roche platform. We found that increasing the TSH threshold from ≥4.50 mIU/L to ≥6.50 mIU/L might improve the accuracy of SCH diagnosis and thus possibly guide better patient monitoring and management, which means a new TSH threshold should be identified according to the real TSH distribution characteristics in Chinese population.

## Data Availability Statement

The raw data supporting the conclusions of this article will be made available by the authors, without undue reservation.

## Ethics Statement

The studies involving human participants were reviewed and approved by the Chinese People’s Liberation Army General Hospital ethics committee. The patients/participants provided their written informed consent to participate in this study.

## Author Contributions

QZ designed this study. Y-sZ wrote the original drafts. S-yD reviewed and edited the manuscript. YG acquired and analyzed the data. J-hW and FW wrote the review and prepared the tables. Y-sZ and S-yD contributed equally as co-first authors. All authors read and approved the final manuscript. We agree to the terms of the BioMed Central Copyright and License Agreement.

## Conflict of Interest

The authors declare that the research was conducted in the absence of any commercial or financial relationships that could be construed as a potential conflict of interest.

## Publisher’s Note

All claims expressed in this article are solely those of the authors and do not necessarily represent those of their affiliated organizations, or those of the publisher, the editors and the reviewers. Any product that may be evaluated in this article, or claim that may be made by its manufacturer, is not guaranteed or endorsed by the publisher.
